# Systemic resistance in citrus to *Tetranychus urticae* induced by conspecifics is transmitted by grafting and mediated by mobile amino acids

**DOI:** 10.1093/jxb/erw335

**Published:** 2016-09-28

**Authors:** Blas Agut, Jordi Gamir, Josep A. Jaques, Victor Flors

**Affiliations:** ^1^Unitat Associada d’Entomologia IVIA-UJI, Departament de Ciències Agràries i del Medi Natural, Universitat Jaume I (UJI), Campus del Riu Sec, E-12071-Castelló de la Plana, Spain; ^2^Metabolic Integration and Cell Signalling Group, Departament de Ciències Agràries i del Medi Natural, Universitat Jaume I (UJI), Campus del Riu Sec, E-12071-Castelló de la Plana, Spain; ^3^Unit of Plant Biology, Université de Fribourg, Avenue de l’Europe 20, 1700 Fribourg, Suïssa

**Keywords:** Citrus, glutamate-receptor like, glutamic acid, grafting, systemic resistance, *Tetranychus urticae.*

## Abstract

Recent research suggests that systemic signalling and communication between roots and leaves plays an important role in plant defence against herbivores. In the present study, we show that the oviposition of the two-spotted spider mite *Tetranychus urticae* in the systemic leaves of citrus rootstock *Citrus aurantium* (sour orange) was reduced by 50% when a lower leaf was previously infested with conspecifics. Metabolomic and gene expression analysis of the root efflux revealed a strong accumulation of glutamic acid (Glu) that triggered the expression of the citrus putative glutamate receptor (*GRL*) in the shoots. Additionally, uninfested sour orange systemic leaves showed increased expression of glutamate receptors and higher amounts of jasmonic acid (JA) and 12-oxo-phytodienoic acid in plants that were previously infested. Glu perception in the shoots induced the JA pathway, which primed *LOX-2* gene expression when citrus plants were exposed to a second infestation. The spider mite-susceptible citrus rootstock Cleopatra mandarin (*C. unshiu*) also expressed systemic resistance, although the resistance was less effective than the resistance in sour orange. Surprisingly, the mobile signal in Cleopatra mandarin was not Glu, which suggests a strong genotype-dependency for systemic signalling in citrus. When the cultivar Clemenules (*C. clementina*) was grafted onto sour orange, there was a reduction in symptomatic leaves and *T. urticae* populations compared to the same cultivar grafted onto Cleopatra mandarin. Thus, systemic resistance is transmitted from the roots to the shoots in citrus and is dependent on rootstock resistance.

## Highlight

Systemic resistance in citrus against *Tetranychus urticae* is rootstock dependent and is transmitted by grafting. Roots release glutamic acid after infestation, inducing *GRL* expression and promoting resistance in the canopy.

## Introduction

Plants react to a herbivore attack by locally activating physical or chemical defensive barriers around injured tissues. Depending on the nature of the herbivore, each reaction may be different. The appropriate recognition of the attacker is relevant to the survival of the host. There are receptors responsible for herbivore recognition through herbivore-associated molecular patterns (HAMPs) and damage-associated molecular patterns (DAMPs) in the cell membrane. Once a herbivore is recognized, a cascade of events is triggered. These defence events typically involve activation of jasmonic acid (JA) signalling and the synthesis of secondary metabolites that have detrimental effects on the herbivore (Kant *et al.*, 2004; Agut *et al.*, 2014; Zhurov *et al.*, 2014). A subsequent defence mechanism consists of the activation and synthesis of toxic compounds that hamper herbivore development or reproduction in distal undamaged plant parts. This mechanism is called systemic resistance (SR). This phenomenon has been largely studied in plant–microbe interactions called systemic acquired resistance. For a long time, the systemic signal transported to undamaged plant parts was unknown. Recently, though, some compounds have been identified as mobile signals that could be responsible for the systemic induction of salicylic acid (SA) in distal plant parts, including methyl salicylate, azelaic acid, dehydroabietinal, glycerol-3-phosphate, and pipecolic acid (Park *et al.*, 2007; Vlot *et al.*, 2009: Jung *et al.*, 2009; Liu *et al.*, 2010; Manosalva *et al.*, 2010; Chanda *et al.*, 2011; Chaturvedi *et al*., 2012; Návarová *et al.*, 2012). In herbivorous arthropods, these direct defence mechanisms can interfere with their reproduction and/or behaviour. In addition, plants can indirectly defend themselves through the synthesis of volatile organic compounds that attract predators of the attacking herbivore (Bruinsma *et al.*, 2009; Mithöfer and Boland, 2012; Huffaker *et al.*, 2013). JA has been implicated in both direct and indirect plant defence mechanisms. Moreover, the role of JA in the systemic defence response to herbivore attack is widely acknowledged in the literature (Schilmiller and Howe, 2005; Heil and Ton, 2008). The role of the oxylipins has previously been described, as has their link with abscisic acid (ABA) in the regulation of systemic responses primed by JA-dependent defences against herbivores (Vos *et al.* 2013). There are other factors influencing anti-herbivore defence, such as vascular architecture. Plant architecture produces spatial variations in the induction of invertases and phenolic compounds (Ferriere *et al.*, 2015). Another important component in SR to herbivore attack is the levels of cytoplasmic calcium ([Ca^2+^]cyt). *Spodoptera littoralis* Boisduval (Lepidoptera: Noctuidae) feeding on Arabidopsis induces increases in both local and systemic [Ca^2+^]cyt (Kiep *et al.*, 2015). Higher levels of [Ca^2+^]cyt are also induced in adjacent leaves with direct vascular connections, which indicates that [Ca^2+^]cyt is increased irrespective of the vascular connectivity.

Evidence of SR to herbivores has been observed in several plant–arthropod systems. Initial evidence of herbivore-induced SR was provided by Karban and Carey (1984). These authors showed that cotton (*Gossypium hirsutum* L.) seedlings previously exposed to *Tetranychus urticae* (Koch) (Acari: Tetranychidae) had lower populations of the mite compared to newly infested plants. This form of SR is mainly related to JA-dependent responses (Heil and Ton, 2008; Soler *et al.*, 2013; Tian *et al.*, 2014). In fact, the induction and expression of proteinase inhibitors in undamaged distal leaves after herbivore attack are controlled by the JA pathway (Howe *et al.*, 1996; Li *et al.*, 2005). However, the identity of the mobile signal responsible for JA activation in the systemic leaves remains controversial. Mousavi *et al.* (2013) implicated glutamate receptors (GRLs) in the systemic response in *Arabidopsis thaliana*. In addition to binding glutamate, these receptors can detect electric changes in the cell surface of wounded cells. Interestingly, these receptors must be intact for functional activation of systemic JA signals in undamaged tissues. Roots have an important role in producing mobile signals that target receptors in distal leaves because there is close communication between aboveground and belowground tissues (Erb *et al.*, 2015). An attack by the root feeder *Diabrotica virgifera* LeConte (Coleoptera: Chrysomelidae) increased resistance in maize (*Zea mays*) against *Spodoptera littoralis* in aboveground tissues (Erb *et al.*, 2008). Maize plants infested with *D. virgifera* showed an accumulation of ABA that was produced in the roots. The accumulation of this hormone altered the water content, which affected the nutritional quality of the leaves for leaf feeders. Conversely, environmental conditions in leaves can produce systemic changes in root physiology.

More evidence for the relevance of the roots was observed in *Nicotiana attenuata* plants. Leaves attacked by herbivores produced an unknown mobile signal that was perceived by the root. Subsequently, this tissue began to produce nicotine, which is a secondary compound with insecticidal activity (Wu and Baldwin, 2010). Using *N. attenuata* plants with impaired JA signalling in the roots, researchers demonstrated that a functional JA pathway in the roots was necessary to sustain a correct defence response in the leaves (Fragoso *et al.*, 2014). Notably, local production of leaf JA and ABA and some shoot metabolites is regulated by JA from the roots. In maize plants attacked by *Spodoptera frugiperda* (J.E. Smith), there is upregulation of the JA pathway and an increase in a specific set of defence proteins in local tissue. In this system, JA was proposed to be the systemic signal travelling through the vascular connections to the roots. Increased levels of JA in roots resulted in a large accumulation of maize insect resistance 1-cysteine protease, which can be translocated to the leaves to interfere with *S. frugiperda* caterpillars (Ankala *et al.*, 2013).

In a previous study, we showed that Cleopatra mandarin (*Citrus unshiu*) is highly susceptible to this mite and, in contrast, sour orange (*C. aurantium*) showed elevated levels of resistance associated with the oxylipin pathway (Agut *et al.*, 2014). Furthermore, herbivore-induced plant volatiles released by sour orange are able to stimulate induced resistance in Cleopatra mandarin. Therefore, both species retain genetic mechanisms to express induced resistance against this mite (Agut *et al.*, 2015). Despite the rapid adaptation of *T. urticae* to synthetic pesticides, there are families of natural secondary metabolites that remain toxic to *T. urticae*, such as flavonoids and glucosinolates (Agut *et al.*, 2014; Zhurov *et al.*, 2014). JA seems to influence the accumulation of phenylpropanoid phytoalexins, such as naringenin and hesperetin, in citrus. In addition, the release of volatiles with a repellent effect on spider mites and induced resistance properties is probably related to the JA and oxylipin pathways (Bruisma *et al.*, 2009; Agut *et al.*, 2015). Citrus rootstocks provide tolerance to many abiotic stresses, such as salinity or drought, and resistance or tolerance to pathogens, such as *Phytophthora citrophthora* or Citrus tristeza virus. Nevertheless, citrus rootstock breeding has never been based on resistance or tolerance to arthropod pests. In 2010, Bruessow *et al*. showed how rootstock affected the intrinsic rate of increase of *T. urticae* in citrus. The same cultivar grafted onto different rootstocks affected the population densities of the mite. Despite new advances, the level of citrus resistance against spider mites is largely unknown. The events regulated by the belowground tissues of the rootstock remain especially unclear.

In the present research, we have studied systemicallytransmitted resistance in the upper canopy of citrus plants when the bottom leaves are infested by spider mites. We have also investigated how different rootstock-cultivar combinations determine the resistance in grafted cultivars and have examined the nature of mobile signals responsible for the SRs that are released by the rootstocks through the vasculature.

## Materials and methods

### Plant material

In this study we used two different kind of plants: the rootstocks sour orange (*C. aurantium*) and Cleopatra mandarin (*C. unshiu*), and the cultivar Clemenules INIASEL 22 (*C. clementina*) grafted onto either sour orange or Cleopatra mandarin. For rootstock experiments, 12-week-old citrus rootstocks were maintained in a climatic chamber at 25ºC and 50–70% relative humidity under a 16:8h light:dark photoperiod. These plants were grown on vermiculite and peat (1:3, vol:vol). The grafted plants were maintained in a greenhouse located at Universitat Jaume I (UJI) at 22±5°C and 50–70% relative humidity under a natural photoperiod. Plants were grown on a substrate consisting of sand and peat (1:1) in 6L cylindrical containers. The leaves used to maintain our spider mite colony (see below) were obtained from plants of the cultivar Clemenules INIASEL22 grafted onto Citrange Carrizo (*Poncirus trifoliata* × *C. sinensis*), another commonly used citrus rootstock, and were maintained under the same conditions as the other grafted plants. No insecticides or acaricides were applied to these plants, which were fertilized every 3 days using a modified Hoagland’s according to Camañes (2007). For grafting experiments, 2-year-old plants with a fully developed scion were used in the combinations Clementine on Cleopatra mandarin and Clementine on sour orange.

### Spider mite stock colony

The *T. urticae* colony used in the assays was initiated with specimens collected in clementine orchards in the region of La Plana (Castelló, Spain). The colony was maintained on detached leaves of young Clemenules plants. The rearing took place on detached leaf units consisting of a single leaf placed upside down on moistened cotton, placed on top of a water-saturated foam cube (3–4cm thick) in an open plastic box (35×20×7cm) half-filled with water. Moist cotton was folded over the edge of the leaf to prevent mites from escaping. When necessary, cohorts of the same age were produced by transferring gravid females from the stock colony to freshly set detached leaf units for a controlled period of time. Afterwards, females were removed and the eggs were kept undisturbed until reaching the desired target stage and age. These cohorts were maintained under the same environmental conditions as the stock colony.

### Systemic resistance in rootstocks

Cleopatra mandarin and sour orange plants were used in these assays. We infested these plants with 10 adult females and included uninfested control plants. To prevent mite dispersal to distal parts of the plants, a ring of the trunk directly above the infested leaves was painted with Tangle-Trap insect trap coating (Tanglefoot Company, Bozeman, MT, USA). Three days later, clean distal parts of the plants (infested and uninfested controls) were infested with six 2-day-old *T. urticae* females. Three days later the number of eggs per plant was assessed. This experiment was repeated three times.

### Hormonal analyses in systemic resistance experiments

Control and infested plants (sour orange and Cleopatra mandarin) were used for analyses. Three days after infestation of basal leaves with adult mites, the uninfested distal leaves were collected and the hormonal content was analysed. The hormones 12-oxo-phytodienoic acid (OPDA), JA, JA-isoleucine (JA-Ile), ABA, and SA were analysed by ultraperformance liquid chromatography coupled to mass spectrometry (UPLC-MS), as described by Flors *et al.* (2008) and Forcat *et al.* (2008).

### Collection of leaf efflux

Three days after infestation of basal leaves with adult mites, mature leaves were excised by cutting the petioles from the leaf blade under the surface of 5mM 2-Na-ethylenediaminetetracetic acid, disodium salt (EDTA), pH 7.0. The petioles of excised leaves were inserted into 1.5mL Eppendorf tubes with 1.0mL of EDTA solution. Tubes were then placed in a closed chamber under low-light conditions and close to 100% relative humidity to reduce transpiration. After 8h we removed the leaf and kept the liquid solution at −20ºC for metabolomic analysis.

### Response of Clemenules mandarin plants grafted on selected citrus rootstocks to mite attack

We used 2-year-old Clemenules plants grafted on either sour orange or Cleopatra mandarin. The base of the trunk of each plant was painted with Tanglefoot® (Tanglefoot Co., Grand Rapids, MI 49504, USA) to prevent ambulatory mite dispersal between plants. Six plants (replicates) per rootstock-cultivar combination were infested with 20 females taken directly from the stock colony and randomly transferred to each plant with a fine camel paintbrush. For the following 2 weeks, spider mite numbers were scored in three leaves randomly selected per plant. Furthermore, symptomatic leaves per plant (those exhibiting typical chlorotic spots) were also scored. These parameters were checked daily until mite-induced defoliation started.

### Collection of root efflux

Twelve-week-old rootstocks were infested with 20 mites per plant. Three days later, root sap samples were collected by cutting plant stems near the base (5–10mm above the ground) and inserting them (upside down) in a Scholander-type pressure chamber (Plant Moisture Systems, Santa Barbara, CA, USA). The cut end protruded by about 5–8mm through the air-tight rubber compression gland. Pressure was applied by filling the chamber with compressed air. The resultant excreted root sap was immediately collected with a micropipette and stored in a 1.5mL Eppendorf tube. Because between 50 and 100 μL of sap were collected in most cases, samples from three plants were pooled. The collected samples were weighed and diluted with double distilled water (typically 10–20 times), frozen, and kept at −18°C until measured. The assay was replicated three times.

### Quantitative real-time PCR analysis

*GRL* and *LOX2* expression analyses of Cleopatra mandarin, sour orange, and Clemenules grafted on either sour orange or Cleopatra mandarin were performed using the Plant RNA kit (Omega Bio-Tek Inc., Doraville, GA, USA). For quantitative real-time PCR (RT-qPCR) experiments, 1.5 μg of total RNA was digested with 1 unit of DNase (RQ1 RNase-Free DNase) in 1 μL of DNase buffer and Milli-Q water up to 10 μL (Promega Corporation, Madison, WI, USA) and incubated for 30min at 37ºC. After incubation, 1 μL of RQ1 DNase stop buffer was added and incubated again at 65ºC for 10min to inactivate the DNase. The RT reaction was performed by adding 2 μL of RT buffer, 2 μL of 5mM dNTP, 2 μL of 10 μM Oligo(dT) 15 primer (Promega), 1 μL of 10U μL−1 RNase inhibitor (Promega), and 1 μL of Omniscript reverse transcriptase (Qiagen, Barcelona, Spain). The reaction mixture was incubated at 37ºC for 60min. Complementary DNA from the RT reaction, diluted ×10, was used for qPCR. Forward and reverse primers (0.3 μM) were added to 12.5 μL of PCR SYBR reaction buffer, 2 μL of cDNA, and Milli-Q sterile water up to 25 μL of the total reaction volume (Takara Bio, Kyoto, Japan). qPCR was carried out using the Smart Cycler II (Cepheid, Sunnyvale, CA, USA) sequence detector with standard PCR conditions. Because there were differences in cycle numbers during the linear amplification phase between samples, the data were transformed with the formula 2^ΔΔCt^. RT-qPCR analysis was performed at least three times using sets of cDNA samples from independent experiments. The primers of *GRL*, *LOX2*, and the housekeeping genes *GADPH* and *EF1* were used (Agut *et al.*, 2014). The sequence of the *GRL* primers were as follows: left primer GGGGCGGGACATTAAATCTT; right primer CTGCGGATACCCATGTTCAA.

### Metabolome analysis: liquid chromatography interfaced with a quadrupole time-of-flight mass spectrometer and an electrospray ionization source

Metabolome samples from leaf and root efflux were used. Metabolome analysis was performed using an Acquity UPLC system (Waters) interfaced to hybrid quadrupole time-of-flight (QTOF Premier). Three technical and three independent biological replicates per sample were randomly injected. The LC separation was performed on an HPLC SunFire C18 analytical column, 5 μm particle size, 2.1×100mm (Waters). Analytes were eluted with a gradient of methanol and water containing 0.01% HCOOH (methanoic acid). The gradient started with 90% aqueous mobile solvent and linearly reached 10% in 12min. In the following 3min, the gradient was kept in isocratic conditions and then returned to initial conditions in 4min. The column was allowed to equilibrate for 3min, giving a total time of 22min per sample. The solvent flow rate was 0.3mL min^−1^. The injection volume was 20 μL. The drying gas and the nebulizing gas were nitrogen. The desolvation gas flow was set to approximately 600L h^−1^, and the cone gas flow was set to 60L h^−1^. A cone voltage of 20V and a capillary voltage of 3.3kV were used in the negative ionization mode. The nitrogen desolvation temperature was set at 350ºC, and the source temperature was set at 120ºC. The instrument was calibrated in the m/z 50–1000 range with a 1:1 mixture of 0.01M NaOH/1% HCOOH tenfold diluted with CH_3_CN (acetonitrile):H_2_O (80:20, vol:vol). A solution of leucine enkephalin at a concentration of 2ppm in CH_3_CN:H_2_O (50:50, vol:vol) with 0.1% HCOOH was simultaneously introduced into the quadrupole time-of-flight (Q-TOF) instrument via the lock-spray needle for accurate m/z determinations. The [M−H]− ion of leucine enkephalin at m/z 554.2615 was used for recalibrating the m/z axis. Metabolite amounts were analysed on the basis of normalized peak area units relative to the freeze-dried weight. A Kruskal–Wallis test (*P*< 0.05) was applied to test the metabolomic differences between rootstocks versus infestation.

For full-scan data analysis, centroid acquired raw data were transformed into .cdf files using Databridge from the Masslynx 4.1 software (Waters) and subsequently subjected to analysis using the software R for statistical purposes. Signals from positive and negative electrospray analysis (ESI^+^; ESI^−^) were processed separately. Peak peaking, grouping, and signal corrections were developed applying the algorithm XCMX. This statistical package can be used to pre-process full-scan LC/MS data for relative quantification and statistical analysis. The statistics and the heat map analysis were carried out with the MarVis Suit software including MarVis Filter and MarVis Cluster (Kaever *et al.*, 2012), a tool for clustering and visualization of metabolic biomarkers. MarVis was used to process exported CSV. files from the XCMS and to perform statistical analysis, adduct and isotope correction, clustering, and colour heat map visualization. To determine a global behaviour of the signals, principal component analysis (PCA) was used.

### Chemical treatments

Twelve-week-old sour orange plants were watered with 100mM glutamic acid (Glu). Three days later, these plants were infested with six 2-day-old *T. urticae* females. After three additional days, the number of eggs deposited was assessed and leaves were collected for RNA analyses.

### Statistical analysis

Statistical analyses of genetic and metabolomic data were conducted using Statgraphics Plus 3.1 (Rockville, MD, USA) and the software R v.2.9.2 (R Development Core Team), and the package XCMX, respectively. All experiments were repeated at least three times unless otherwise specified.

Mean mite densities and symptomatic leaf counts in the Clemenules cultivar grafted on each rootstock over time in the third assay were compared using a repeated measures generalized linear mixed model (the fixed factor was rootstock and the sampling date was the random factor). When required, data were square root transformed to fulfil the assumption of normality. Our first approach in the variable symptomatic leaves was to use a normal distribution, based on results of Akaike’s information criterion and the distribution of residuals compared to negative binomial and Poisson distributions. In contrast, for the variable mite density we used a gamma distribution, as it is better adapted to the requirements already mentioned.

When significant differences were found, pairwise comparisons of the fixed factor levels were performed with the least significant difference (LSD) *post hoc* test (*P* < 0.05). A minimum of three different biological replicates were performed for each experiment.

## Results

### Both the mite-susceptible Cleopatra mandarin and the resistant sour orange rootstocks produce systemic resistance but to different extents

**Fig. 1.**
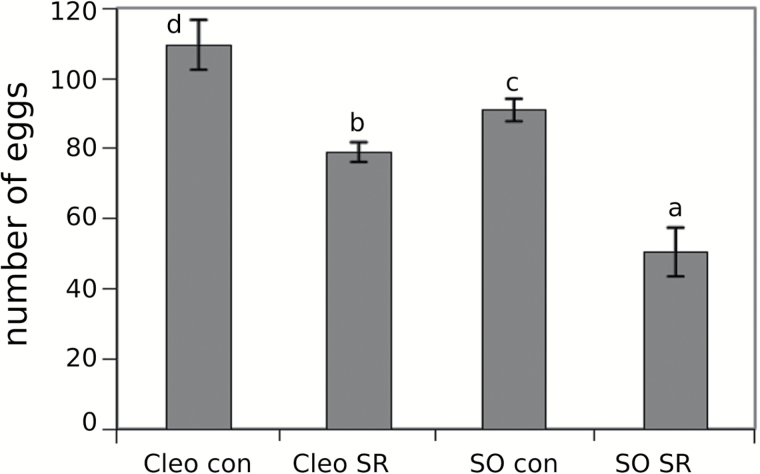
**Effect of SR treatments of Cleopatra mandarin and sour orange on spider mite oviposition rates**. Half of the plants were infested with 10 *T. urticae* adult females (SR treatments). Three days later, all of the plants were infested with 2-day-old *T. urticae* adult females on distal clean leaves. Egg number was determined 3 days after the second infestation. Different letters indicate significant differences between the treatments (ANOVA; LSD test, *P* < 0.05). The figure shows the average results of three biological replicates (*n* = 9). Cleo con, Cleopatra mandarin uninfested; Cleo SR, Cleopatra mandarin previously infested; SO con, sour orange uninfested; SO SR, sour orange previously infested.

The phenomenon of SR following infestation by *T. urticae* was previously demonstrated by Karban and Carey (1984). In the current study, SR against *T. urticae* was studied in rootstocks from both species. We infested the bottom leaves of these rootstocks with 10 mites. Three days later, a second infestation with female mites was performed. Both rootstock species displayed SR in the distal leaves (Fig. 1). However, the level of protection was not the same. SR in sour orange resulted in a 50% reduction in the mite population, whereas the reduction was approximately 30% in Cleopatra mandarin. In conclusion, both genotypes can express SR, but resistance is higher in sour orange than in Cleopatra mandarin.

### Metabolomic analyses revealed candidates responsible for systemic resistance

**Fig. 2.**
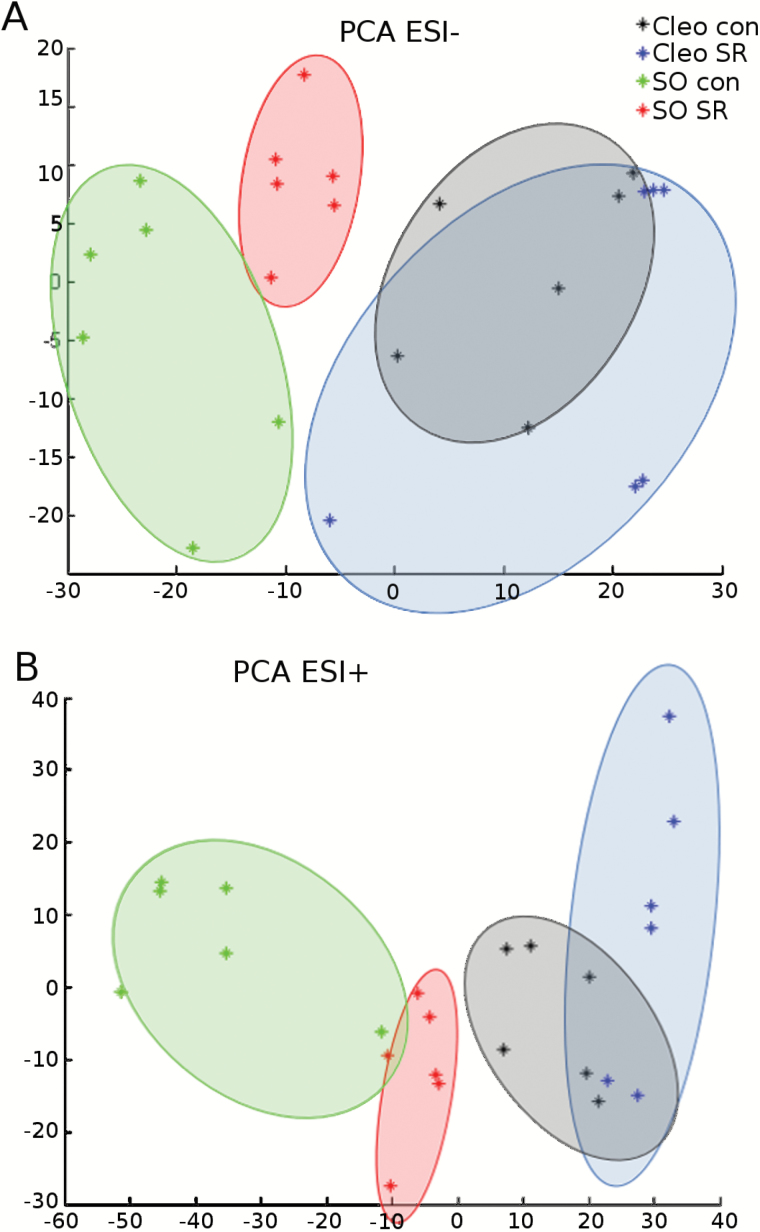
**PCA of the metabolomic fingerprint of sour orange and Cleopatra mandarin leaf efflux following SR treatments and *T. urticae* infestation.** Non-supervised PCA representation of major sources of variability of ESI^−^ (**A**) and ESI^+^ (**B**) signals obtained from a non-targeted analysis by UPLC-Q-TOF-MS to monitor metabolomic changes during spider mite infestation. Four different sets of samples were tested: sour orange uninfested (SO con), sour orange previously infested (SO SR), Cleopatra mandarin uninfested (Cleo con), and Cleopatra mandarin previously infested (Cleo SR). Twelve-week-old plants were infested with 10 mites per plant. Three days later, infested leaves were cut, and the petiole was submerged in an EDTA solution for 8h to collect the leaf efflux. Three independent biological and two technical replicates were randomly injected and analysed (*n* = 6). This figure is available in colour at *JXB* online.

To determine candidates for the signals that are transmitted from infested leaves to distal leaves, we collected sap efflux from locally infested leaves and performed LC-ESI-Q-TOF analysis. Comparative unsupervised PCA showed that infestation explained 18.2% and 16.1% of the total variation for ESI^+^ and ESI^−^ signals, respectively (Fig. 2). The Q-TOF detected approximately 800 signals in each treatment. In the absence of infestation, the metabolic content of the leaf exudate was different between the two rootstocks, and the differences revealed strong basal differences in sap composition (Fig. 2). By comparing both rootstocks after previous infestation, a significant separation of the metabolites for sour orange was observed, whereas an overlap of the pool of compounds was found in Cleopatra mandarin. Therefore, mite infestation strongly modified the composition of the metabolites secreted by the leaves of sour orange. In contrast, changes in the global behaviour of the metabolites were subtler in Cleopatra mandarin although there were signs of SR.

**Fig. 3.**
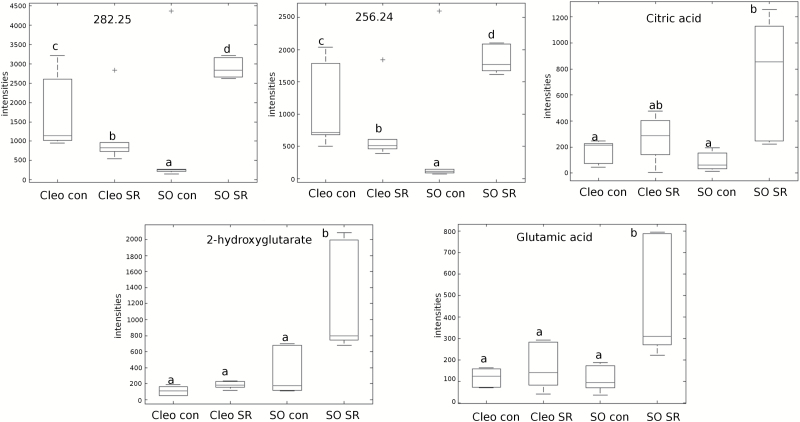
**Signals accumulated in the leaf efflux of SR-treated sour orange.** Citric acid, Glu, 2-hydroxyglutarate, and two oxylipins either fully or tentatively identified by exact mass as: 282.25 (hexadecanoic acid) and 256.24 (octadecanoic acid). Boxplot analysis of the relative abundance of the five compounds in sour orange (SO) and Cleopatra mandarin (Cleo) either in the absence (con) or presence of spider mites (SR). Different letters indicate significant differences between the treatments (ANOVA; LSD test, *P* < 0.05; *n* = 6).

The accurate masses provided by the Q-TOF detector were entered into online biological databases, such as Metlin to match mass identity, and KEGG and AraCyc for a pathway search. Several compounds that were upregulated in previously infested Cleopatra mandarin and sour orange rootstocks were identified (Supplementary Fig. S1 at *JXB* online). One of them was myo-inositol, which was confirmed by matching the spectra with experimental masses and fragmentation obtained from the Metlin database (Supplementary Fig. S2). Three additional tentative masses were identified, including 112.01, 124.28, and 469.39 (Supplementary Fig. S1). These compounds were also found in higher concentrations in plants previously infested with *T. urticae*. Although the two rootstocks showed functional SR, the induction of SR was stronger in sour orange. Therefore, we focused our attention on metabolites that could function as possible warning signals in sour orange SR. Five masses matched the criteria of high accumulation after sour orange plants were infested (Fig. 3). From these masses, we were able to fully identify citric acid and 2-hydroxyglutarate (by matching the exact mass and the fragment spectrum with theoretical fragments from Metlin; Supplementary Fig. S2). Two fatty acids were tentatively identified, octadecanoid acid (256.24) and hexadecanoid acid (282.25) (Fig. 3).

Because amino acids are small, mobile compounds that can be transported systemically, we used a library of commercial standards to analyse their relative quantities in leaf exudate. As shown in Supplementary Fig. S3, the amino-acid profiling in the exudate was clearly influenced by *T. urticae* infestation. Leucine, methionine, phenylalanine, and threonine were found in higher concentrations in the leaf efflux of infested Cleopatra mandarin plants than in uninfested control plants. Interestingly, the set of amino acids that increased in sour orange plants was different. Glu (Fig. 3), tyrosine, and proline were exported in higher amounts when sour orange plants were infested. Therefore, these amino acids from Cleopatra mandarin and sour orange were likely transported following *T. urticae* infestation.

### Glu and its perception are important for systemic resistance in sour orange

**Fig. 4.**
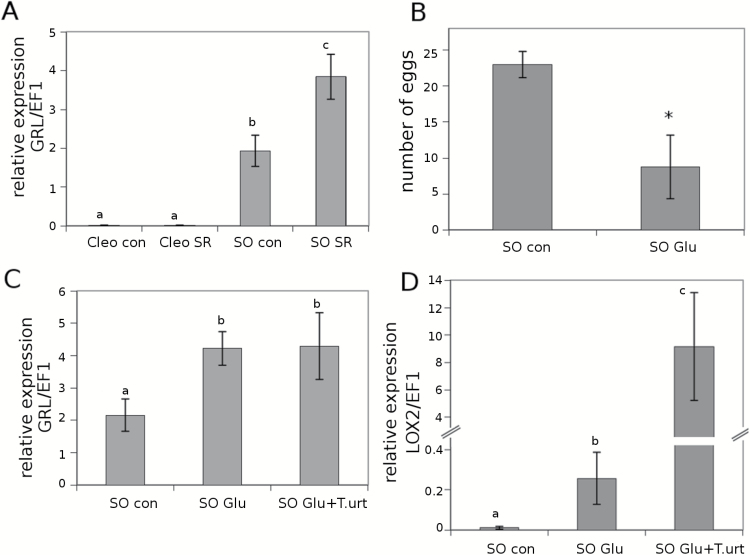
**Relevance of Glu and glutamate receptor-like genes (*GRL*) in SR against *T. urticae* in citrus rootstocks**. Cleopatra mandarin (Cleo) and sour orange (SO) plants were previously infested with 10 *T. urticae* adult females (SR). **(A)** Three days later, distal uninfested leaves of control and SR plants were collected for RT-PCR analysis. Data are presented as a mean of three independent analyses of transcript expression relative to the housekeeping gene plants ± SD (*n* = 3). Different letters indicate significant differences (one-way ANOVA, *P* < 0.05; LSD) between treatments with Ct values as described by Yuan *et al.* (2006). (**B**) Spider mite oviposition in sour orange plants treated with 100mM of Glu (SO Glu) compared with control plants (SO con). Three days after Glu treatment, the plants were infested with 2-day-old *T. urticae* adult females. Three days later, the number of eggs was assessed. The asterisk indicates a significant difference between different treatments (*t*-test; *P* < 0.05). **(C)** *GRL* and **(D)** *LOX2* expression levels in sour orange plants treated with 100mM of Glu (SO Glu). Three days later, the plants were infested with 2-day-old *T. urticae* adult females and 3 days after that the leaves were collected for RT-PCR analysis. Data are presented as a mean of three independent analyses of transcript expression relative to the housekeeping gene plants ± SD (*n* = 3). Different letters indicate significant differences (one-way ANOVA, *P* < 0.05; LSD) between treatments with Ct values as described by Yuan *et al.* (2006).

Glutamate receptor-like proteins (GRLs) are involved in leaf-to-leaf transport communication and provide information about the status of neighbouring leaves after the plant is damaged (Mousavi *et al.*, 2013). Furthermore, these receptors seem to promote JA-dependent signals in distal undamaged leaves. In fact, GRL Arabidopsis mutants are not able to activate the JA pathway in distal leaves in response to insect attack. As we showed previously, Glu is accumulated in the exudate of leaves from sour orange plants upon mite infestation. Homologous *GRL* expression was tested in the systemic leaves of infested citrus plants (Fig. 4A). Interestingly, *GRL* expression was strongly induced in systemic uninfested sour orange leaves, whereas *GRL* expression was hardly detectable in Cleopatra mandarin. This result implies Glu and citrus GRLs are involved in sour orange SR, whereas it remains unclear which signals are mediating SR in Cleopatra mandarin. To determine whether Glu is the mobile signal inducing SR in sour orange, experiments involving Glu treatment prior to infesting the plants with *T. urticae* were conducted. The Glu treatments clearly reduced spider mite oviposition and boosted the expression levels of *GRL* and *LOX2* (Fig. 4B–D). Oviposition was reduced by 60% in the Glu-treated plants (Fig. 4B). *GRL* expression was upregulated in Glu-treated sour orange plants compared to control plants independently of the presence of mites (Fig. 4C). Glu acid treatments primed the expression of *LOX2*. This gene was induced somewhat by Glu treatments and strongly induced following spider mite infestation (Fig. 4D). This result suggests that Glu treatment conditions the plant for a stronger and faster response to future attacks.

### Activation of the oxylipin pathway in systemic leaves is linked to systemic resistance in sour orange and may be related to ABA signalling in Cleopatra mandarin

**Fig. 5.**
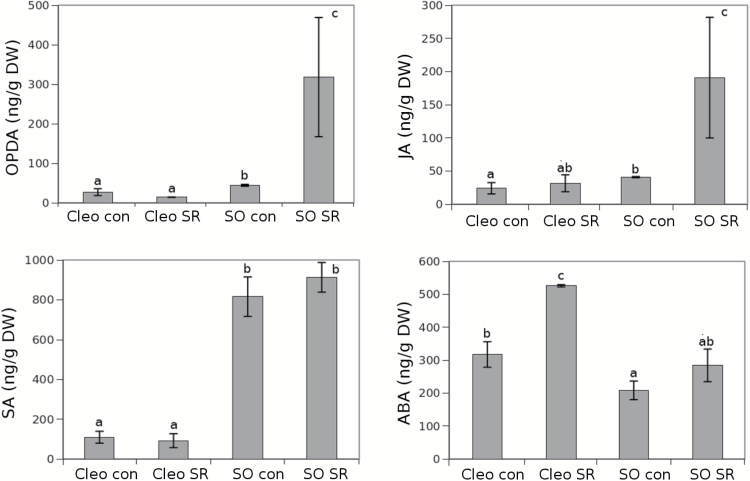
**Hormone levels in sour orange (SO) and Cleopatra (Cleo) mandarin after SR treatment.** Citrus rootstocks were infested with 10 *T. urticae* adult females. Three days later, uninfested distal leaves of these plants were collected to measure hormone levels. JA, OPDA, SA, and ABA levels were determined in freeze-dried material with targeted HPLC-MS. The results shown are the mean of hormone levels from three independent biological replicates ± SD (*n* = 3). Different letters indicate significant differences (one-way ANOVA, *P* < 0.05; LSD) between treatments. Cleo con, Cleopatra mandarin uninfested; Cleo SR, Cleopatra mandarin previously infested; SO con, sour orange uninfested; SO SR, sour orange previously infested.

To determine the influence on the main defence pathways regulated by hormones in systemic responses against *T. urticae*, the hormonal content in uninfested systemic leaves of systemic-response treated plants was analysed (Fig. 5). In SR-induced sour orange plants, the oxylipin hormones OPDA and JA showed a high accumulation in distal clean leaves compared to control plants. Contrastingly, Cleopatra mandarin plants did not show any significant change either in OPDA or JA accumulation. Despite no increase in oxylipin in Cleopatra mandarin, SR still induced a reduction in oviposition, which suggests that a systemic-response is also transmitted in this case. Only ABA increased in systemic leaves of Cleopatra mandarin sysremic-response treated plants plants suggesting that ABA may play a role in this rootstock in the systemic signalling. SA was not altered by systemic-response treatments in any of the two genotypes considered here. We also determined the levels of *PR5* and *ABA4* expression, which are SA and ABA marker genes, respectively (Supplementary Fig. S4). Although there is no statistical correlation, a trend can be observed that explains the changes in hormone levels. These differences could be explained on a time course basis because sampling at earlier time points could fit better with the SA and ABA levels observed (Fig. 5).

### The systemic-induced resistance in sour orange is transmitted by grafting

Rootstocks are commonly used in agriculture because the grafted commercial scions have better performance in terms of tolerance to different stresses and agronomic characteristics. In the present study, we also observed that sour orange transmitted SR more efficiently to uninfested distal leaves, and this phenomenon is likely triggered by Glu and mediated by the oxylipin pathway. Although this phenomenon was also present in Cleopatra mandarin, it appeared to be regulated by different mechanisms. In both cases, the transmitted signals were generated at the bottom part of the plant that had been exposed to *T. urticae*. In the following experiments, we examined whether the resistance observed in the different rootstocks could be transmitted to grafted scions. We used a commercial cultivar of mandarin, Clemenules, as the scion for the rootstocks sour orange and Cleopatra mandarin.

**Fig. 6.**
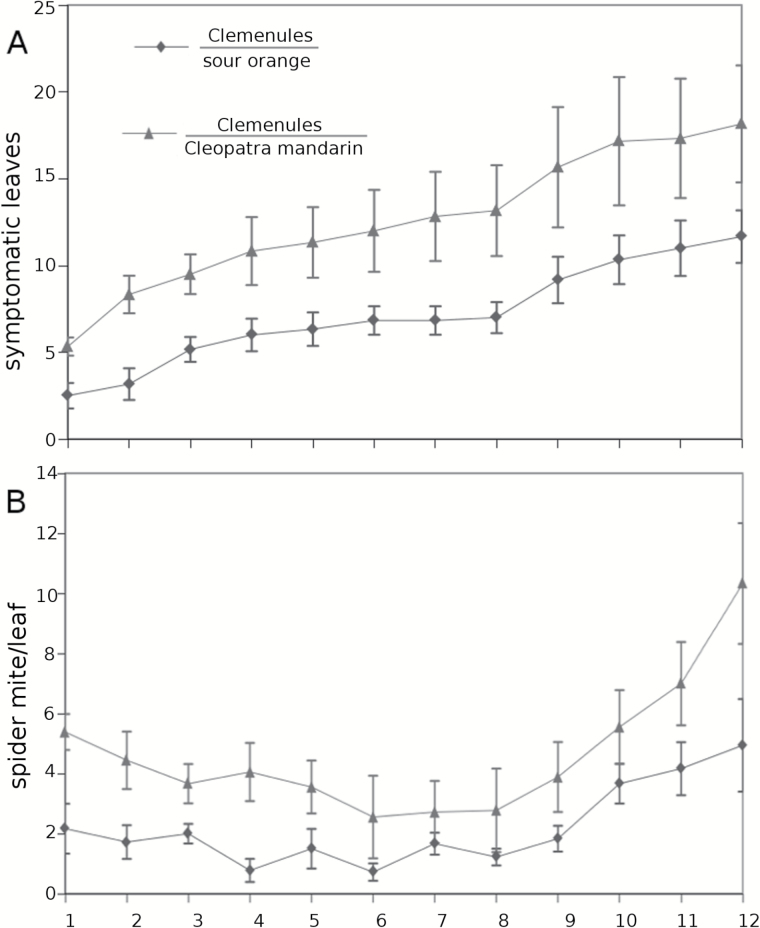
**(A)** Symptomatic leaves and **(B)** spider mites in the Clemenules variety grafted onto sour orange or Cleopatra mandarin rootstocks. 2-year-old plants were infested with 20 *T. urticae* adult females. The samples were collected after the emergence of the first chlorotic leaves until the first symptoms of defoliation. The statistical analyses were conducted with a generalized linear mixed model. This figure is available in colour at *JXB* online.

Two-year-old plants containing the aforementioned combinations of scion-rootstock were infested with 20 adult *T. urticae* females. The number of chlorotic leaves and the number of adult females per leaf was assessed for 2 weeks. Both parameters showed the same trend. The combination Clemenules-sour orange displayed enhanced resistance with lower mite populations and reduced injury levels (chlorotic leaves) compared to Clemenules grafted onto Cleopatra mandarin (Fig. 6).

### Metabolic changes in the scion induced by spider mite infestation are rootstock dependent

**Fig. 7.**
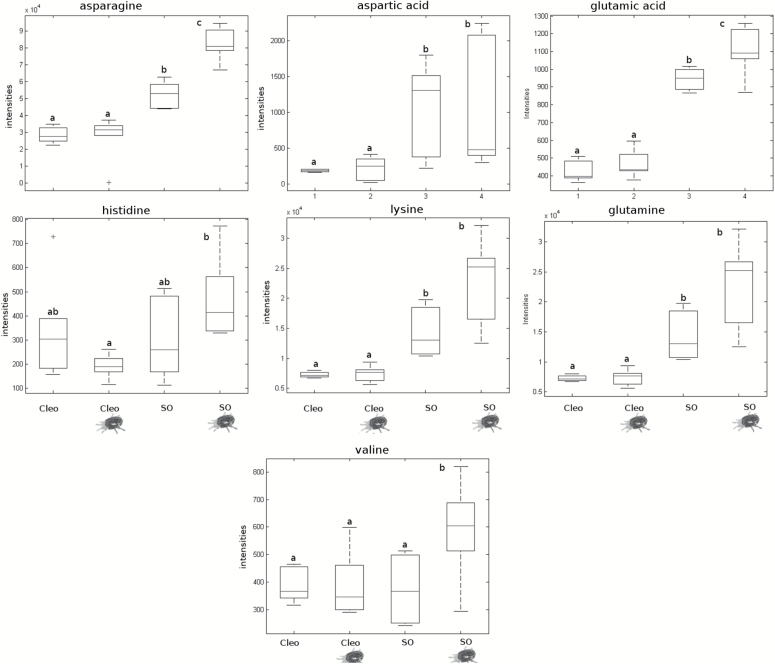
Amino acid profile in the root efflux from rootstocks following spider mite infestation. Clemenules variety grafted onto sour orange (SO) and Clemenules variety grafted onto Cleopatra mandarin (Cleo) plants were either uninfested or infested (mite cartoon). 2-year-old grafted plants were infested with 20 mites per plant. Three days later, the stem was cut and the root efflux was collected using a Scholander pressure chamber. The samples were quantified by HPLC-Q-TOF-MS and processed using an amino acid library. Boxplots represent the average of three independent experiments with two technical replicates (*n* = 6). Different letters indicate significant differences (one-way ANOVA, *P* < 0.05; LSD) between treatments.

To identify the mobile signal that is transmitted from the roots to the leaves, the root efflux from infested plants was collected and analysed by LC-Q-TOF. We did not observe any relevant change in JA or OPDA measured in the root efflux of infested plants compared to their respective controls (Supplementary Fig. S5). As described by Agut *et al.* (2014), the basal level of JA in the absence of infestation was higher in sour orange than in Cleopatra mandarin. However, JA levels were reduced in sour orange following infestation. Subsequently, we identified signals corresponding to different amino acids (Fig. 7; Supplementary Fig. S6). None of the amino acids identified in Cleopatra mandarin changed significantly. In contrast, asparagine, valine, and Glu levels increased in infested sour orange plants. Again, Glu functioned as a key metabolite in sour orange resistance and was very likely the transported signal. We were unable to detect any amino acids not mentioned in the results in our experimental conditions, despite the use of chemical standards.

**Fig. 8.**
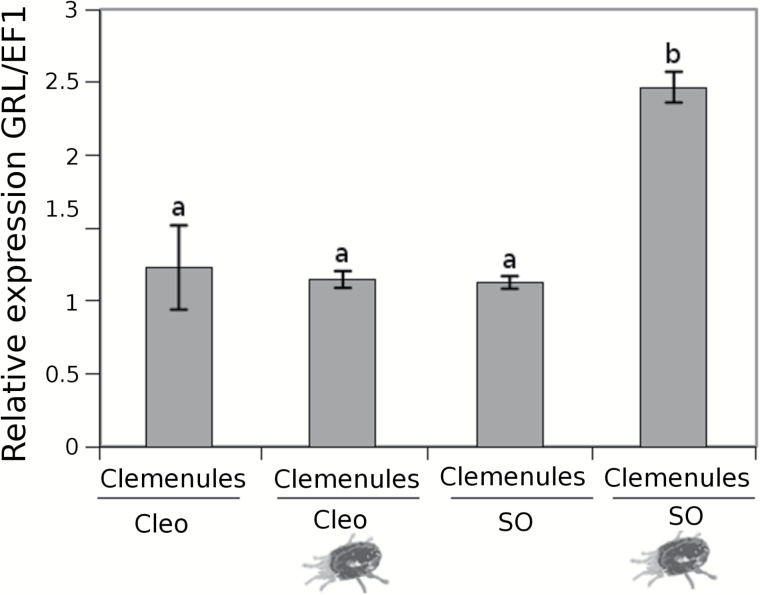
GRL expression in grafted plants affected by spider mites. Clemenules variety grafted onto sour orange (SO) and Clemenules variety grafted onto Cleopatra mandarin (Cleo) plants were either uninfested or infested (mite cartoon). 2-year-old grafted plants were infested with 20 mites per plant. Three days later, leaves were collected for mRNA analysis. Data are presented as a mean of three independent analyses of transcript expression relative to the housekeeping gene plants ± SD (*n* = 3). Different letters indicate significant differences (one-way ANOVA, *P* < 0.05; LSD) between treatments with Ct values as described by Yuan *et al.* (2006).

**Fig. 9.**
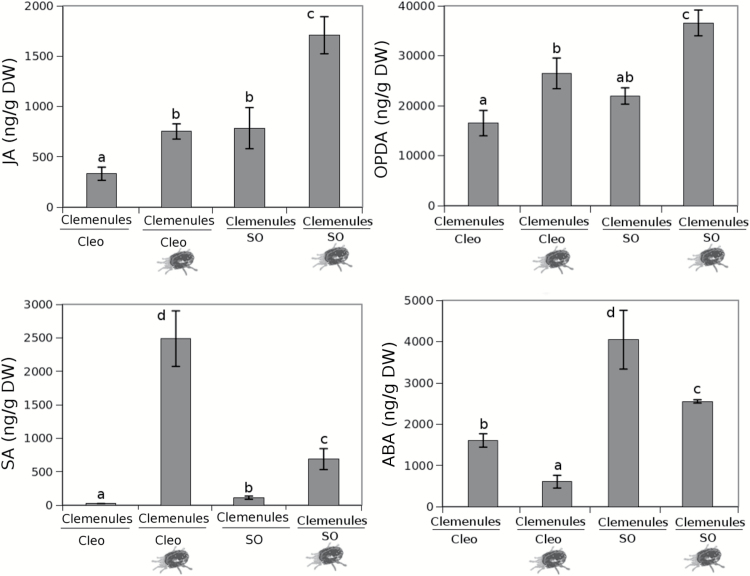
Hormonal content of grafted plants affected by the spider mites. Clemenules variety grafted onto sour orange (SO) and Clemenules variety grafted onto Cleopatra mandarin (Cleo) plants were either uninfested or infested (mite cartoon). The plants were infested with 20 mites per plant. Three days later, JA, OPDA, SA, and ABA levels were determined in freeze-dried material by HPLC-MS. The results shown are mean hormone levels of three independent analyses ± SD (*n* = 3). Different letters indicate significant differences (one-way ANOVA, *P* < 0.05; LSD) between treatments.

Leaf material from Clemenules grafted onto sour orange and Cleopatra mandarin was collected after infestation for further analysis. Despite the leaves belonging to the same genotype, the expression of *GRL* strongly varied in this rootstock (Fig. 8). The Clemenules cultivar grafted onto sour orange showed increased *GRL* expression, which likely occurred owing to the elevated concentrations of Glu in the root flow that triggered *GRL* gene expression more efficiently. Nevertheless, contributions from other amino acids cannot be fully ruled out because Arabidopsis *GRL* may bind other amino acids (Mousavi *et al.*, 2013). As we showed in Fig. 4, Glu increased resistance in sour orange plants by triggering enhanced *LOX2* expression. To determine whether this mechanism was also present in the scion, the hormonal content in Clemenules leaves grafted onto sour orange or Cleopatra mandarin was analysed. Figure 9 shows that Clemenules grafted onto sour orange showed a high accumulation of OPDA and JA after infestation compared to uninfested plants. In Clemenules grafted onto Cleopatra mandarin, there was an increased amount of both oxylipins. However, the increase was weaker than that in Clemenules grafted onto sour orange. Additionally, infestation with *T. urticae* increased the levels of SA in both scion/rootstock combinations. Nevertheless, the accumulation was very strong in Clemenules grafted onto Cleopatra mandarin after infestation. In the case of ABA, infestation with the spider mite reduced levels of this hormone in Clemenules grafted onto either rootstock (Fig. 9).

## Discussion

Following biotic attack, locally damaged tissues send a warning signal to undamaged distal leaves to prepare a defence against subsequent attacks. Following detection of the attack, some signals must travel to the roots, which coordinate subsequent responses. In the present study, the SR of two citrus genotypes was studied. Both the mite-susceptible Cleopatra mandarin and the mite-resistant sour orange rootstocks are able to express SR to a different extent. We identified several accumulated compounds, such as myo-inositol, in the leaf efflux of both genotypes following a spider mite infestation. This sugar and its derivatives are crucial compounds for development and signalling in plants. These molecules participate in the stress response, hormone sensing, and nutrient perception, contributing to stress tolerance in plants (Valluru and Van den Ende, 2011). Nevertheless, the resistant sour orange rootstock showed a stronger SR compared to the susceptible Cleopatra mandarin (Fig. 1). Higher levels of three metabolites – citric acid, 2-hydroxyglutarate, and Glu – accumulated in infested sour orange but not in Cleopatra mandarin, which suggests their involvement in a stronger SR response. These compounds are metabolically linked to the tricarboxylic acid cycle (TCA). Sour orange with previous contact with the mite reorganizes its metabolic fingerprint to a more active TCA cycle, thereby triggering the biosynthesis of Glu as a mobile signal released by the infested leaves to distal parts of the plant. Several mobile signals that trigger the defence response in undamaged tissue after pathogen infection were recently identified in different plant species. Methyl SA, azelaic acid, pipecolic acid, dehydroabietinal, and the lipid transfer protein DIR1 were shown to mediate the SR against different biotic stressors (Park *et al.*, 2007; Vlot *et al*., 2008; Jung *et al.*, 2009; Liu *et al.*, 2010; Manosalva *et al.*, 2010). These compounds play a cooperative role to activate systemic acquired resistance. The role of the TCA cycle in induced resistance and recruitment of beneficial organisms has previously been suggested (Rudrappa *et al.*, 2008; Pastor *et al.*, 2014). Green and Ryan (1973) described the accumulation of proteinase inhibitors in distal leaves after wounding or herbivory in tomato (*Solanum lycopersicum*) leaves. More recently, it has been discovered that membrane depolarization is a critical step for signal transmission (Mousavi *et al.*, 2013). The GRL protein is sensitive to this depolarization and activates the downstream expression of JA marker genes, which leads to the synthesis of toxic compounds with insecticidal activity. The relationship between GRL and JA was reported in Arabidopsis plants overexpressing a radish (*Raphanus sativus*) GRL. These plants displayed enhanced resistance to a fungal pathogen owing to upregulation of JA-responsive and JA-biosynthetic genes (Kang *et al.*, 2006).

In our experimental system, the systemic defence response triggered by first contact with the spider mite was retained by both rootstocks, although it was quantitatively stronger in sour orange, which also displays a stronger basal resistance than Cleopatra mandarin (Agut *et al.*, 2014; Agut *et al.*, 2015). Surprisingly, the susceptible rootstock still retained the ability to express SR but we did not observe a change in Glu levels in the root efflux or any JA activation in distal leaves. It is likely that the first infestation triggers alternative defences in distal plant parts that prime Cleopatra mandarin for subsequent attacks. However, the signals that are transmitted from belowground have yet to be elucidated. The susceptible rootstock Cleopatra mandarin is likely not able to respond to mite attack by activating the JA signalling pathway (Agut *et al.*, 2014) owing to the manipulation of host defences by the mite. However, the undamaged distal leaves of previously infested Cleopatra mandarin showed a strong accumulation of ABA. Previously, Erb *et al.* (2008) described the negative effects of ABA accumulation on the growth of *Spodoptera littoralis*. It is a tempting hypothesis, yet it is rather unlikely that ABA acts as a mobile signal triggered in the roots of Cleopatra mandarin following spider mite infestation because ABA has previously been shown in maize as a response to belowground attack (Erb *et al.*, 2009). We were unable to detect significant changes in ABA levels in infested leaf efflux. Additionally, ABA was reduced in the root efflux. Finally, a significant SA increase was detected in the leaves of Clementine grafted onto Cleopatra mandarin, which supports the antagonistic effect on SA and ABA that has been described before (Yasuda *et al.*, 2008; Pieterse *et al.*, 2009).

In a more detailed study of the mobile signals from the resistant sour orange genotype, Glu was the most likely candidate to stimulate SR in distal undamaged tissues. Kuć (2001) previously proposed Glu as a likely mobile signal triggering SR. The free Glu in the plant could be recognized by the GRL proteins in undamaged distal leaves and start a signal transduction to activate the JA-dependent pathway. To test this possibility in our experimental system, sour orange plants were watered with Glu and subsequently infested with *T. urticae* adult females. Interestingly, Glu treatments slightly increased *LOX2* expression in comparison to water treatment of the control. However, following spider mite attack, *LOX2* expression was induced dramatically by up to 40 times that in the control plants. Notably, this *LOX2* expression follows a defence priming pattern in which the priming stimulus is Glu and the challenge leads to a much stronger response (Balmer *et al.*, 2015).

Roots were historically considered to be largely a support tissue that functions mainly in water and nutrient uptake from the soil, but this oversimplified vision has changed. Recent studies demonstrate that roots perceive and deliver signals to the shoots to enhance and coordinate defensive responses against aboveground insects such as *S. littoralis* (Erb *et al.*, 2008). Wu and Baldwin (2010) proposed that roots can manufacture toxic metabolites. After wounding by lepidopteran larvae, *Nicotiana* plants send an undetermined signal inducing the biosynthesis of the toxic secondary metabolite nicotine to the roots. Recently, Fragoso *et al.* (2014) showed that proper function of the JA pathway in the roots is necessary. Notably, JA produced in the roots regulates aboveground JA levels. Indeed, wild-type tomato plants grafted onto JA mutants showed increased damage from *Empoasca* spp. leafhoppers and *Tupiocoris notatus* Distant (Hemiptera: Miridae). In a clear demonstration of the role of roots against leaf insect attack, Louis *et al.* (2015) demonstrated that foliar feeding by aphids triggers root accumulation of the *maize insect resistance1* gene product that is a potential long-distance signal against aphids.

Bruessow *et al.* (2010) demonstrated the influence of the rootstock on the performance of *T. urticae* in a Satsuma mandarin scion. In the present study, evidence of a systemic signal travelling from the roots to the shoots is provided. The same citrus cultivar Clementine used as a scion grafted onto a susceptible and a resistant rootstock modified the performance of *T. urticae.* In addition to the increased levels of OPDA and JA in systemic leaves of SR-induced plants, it is likely that mobile oxylipin was responsible for the systemic defence in sour orange plants. Surprisingly, the JA levels in the root efflux after *T. urticae* infestation in sour orange were lower than those in the sour orange control plants, which makes this hypothesis unlikely. Interestingly, amino-acid profiling was substantially modified in the root efflux of sour orange and Glu levels were much higher. Again, all our experimental observations point to Glu as the mobile signal that induced SR. Accordingly, enhanced GRL expression in the Clemenules cultivar grafted onto sour orange supported lower densities of *T. urticae* and presented fewer symptomatic leaves than the same cultivar grafted onto Cleopatra mandarin. Mousavi *et al.* (2013) correlated activation of the JA pathway with the GRL receptors because Arabidopsis GRL mutants were not able to induce JA in the systemic leaves. To test whether citrus may resemble this model plant, the hormonal content in the Clemenules cultivar grafted onto sour orange and Cleopatra mandarin was determined. There was a good correlation between the high levels of Glu in the root efflux with higher levels of GRL expression in the leaves of the scion as well as higher levels of JA and OPDA in Clemenules grafted onto sour orange following infestation with *T. urticae.* Accordingly, Clemenules grafted onto Cleopatra mandarin displayed high levels of infestation together with lower levels of OPDA, JA, and GRL expression compared to the combination Clemenules/sour orange. These results clearly show that citrus resistance against spider mites can be transmitted though grafting and the resistance is rootstock dependent.

**Fig. 10.**
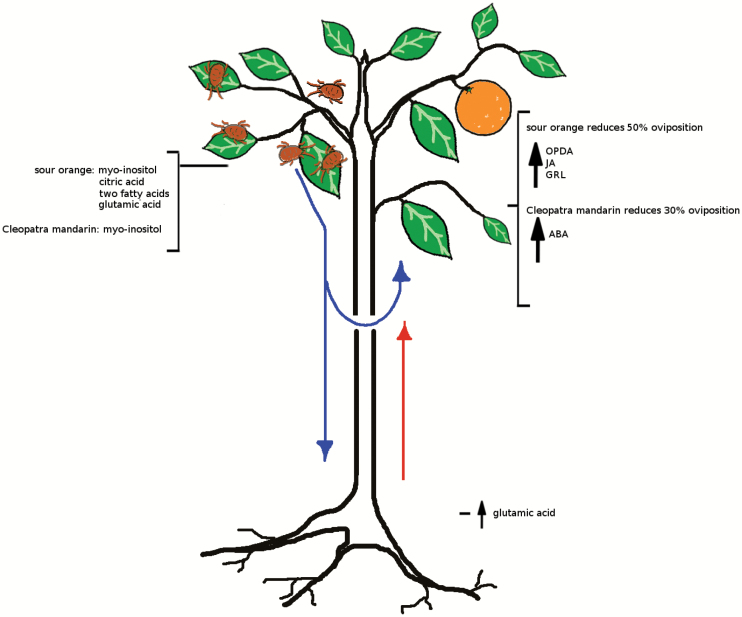
Model for SR against *T. urticae* in citrus. Spider mite attack rapidly induces changes in the leaf efflux. The two rootstocks respond differently to mite infestation. Leaf efflux from Cleopatra mandarin contains high amounts of myo-inositol, whereas sour orange also releases citric acid, Glu, and two fatty acids. These compounds can move to distal leaves or to the root. Once the roots detect the signals from the infested leaves, the resistant rootstock, sour orange, increases the transport of Glu to the shoot. The distal leaves receive the signals from the roots and/or from the infested leaves and respond to a future attack. Consequently, sour orange increases the expression of GRL that activates the JA pathway (high levels of OPDA and JA) and reduces the oviposition of *T. urticae* by 50%. The reduction of *T. urticae* oviposition in Cleopatra mandarin is 30%, possibly due to an increase in ABA levels. (This figure is available in colour at *JXB* online).

In conclusion, defence responses in sour orange against spider mites are perceived and orchestrated by roots that release Glu, among other systemic signals, and activate *GRL* expression, which triggers JA-dependent defences (Fig. 10). In addition, citrus shoot defences vary in a rootstock-dependent manner. Whereas leaf efflux from Cleopatra mandarin contains high amounts of myo-inositol, sour orange efflux also contains citric acid, Glu, and two additional fatty acids. The shoot perceives the attack through an unknown mechanism that is transmitted to the root. Accordingly, the root delivers compounds that move to distal leaves to orchestrate an effective defence response. This response is transmitted through grafting and reduces the oviposition of *T. urticae* by 50% when the scion is grafted on a resistant rootstock.

## Supplementary material

Supplementary data are available at *JXB* online.

Figure S1. Signals accumulated in the leaf efflux of sour orange and Cleopatra mandarin following infestation.

Figure S2. Identification of overaccumulated compounds.

Figure S3. Amino acid profile in the leaf efflux in sour orange and Cleopatra mandarin following spider mite infestation.

Figure S4. *PR5* and *ABA4* expression in in sour orange (SO) and Cleopatra (Cleo) mandarin after SR induction

.

Figure S5. Hormonal profile in the root efflux in grafted plants following spider mite infestation.

Figure S6. Amino acids showing non-significant changes in the root efflux from rootstocks following spider mite infestation.

Supplementary Data

## Abbreviations:

Glu: glutamic acid
GRL: glutamate receptor-like
SR: systemic resistance
JA: jasmonic acid
SA: salicylic acid
ABA: abscisic acid
OPDA: oxo-phytodienoic acid;
ESI: electrospray ionization
TCA: tricarboxylic acid cycle
SO: sour orange
Cleo: Cleopatra mandarin.
