# Supplementary material for: Systemic resistance in citrus to Tetranychus urticae induced by conspecifics is transmitted by grafting and mediated by mobile amino acids
Source: J Exp Bot. 2016 Sep 28;67(19):5711–23. doi: 10.1093/jxb/erw335 (PMC5066491; doi:10.1093/jxb/erw335)
Supplement: Supplementary Data [file supp_67_19_5711__index.html]

Systemic resistance in citrus to Tetranychus urticae induced by conspecifics is transmitted by grafting and mediated by mobile amino acids — Systemic resistance in citrus to Tetranychus urticae induced by conspecifics is transmitted by grafting and mediated by mobile amino acids — Supplementary Data 

# Systemic resistance in citrus to *Tetranychus urticae* induced by conspecifics is transmitted by grafting and mediated by mobile amino acids

## Supplementary Data

Data files

- Supplementary\_figures\_S1\_S6.pdf - Supplementary Data
