# Supplementary material for: Systemic resistance in citrus to Tetranychus urticae induced by conspecifics is transmitted by grafting and mediated by mobile amino acids
Source: J Exp Bot. 2016 Sep 28;67(19):5711–23. doi: 10.1093/jxb/erw335 (PMC5066491; doi:10.1093/jxb/erw335)
Supplement: Supplementary Data [file supp_erw335_Supplementary_figures_S1_S6.pdf]

Systemic resistance to *Tetranychus urticae* induced by conspecifics is transmitted by grafting and mediated by mobile amino acids

Blas Agut, Jordi Gamir , Josep A. Jaques, Victor Flors.

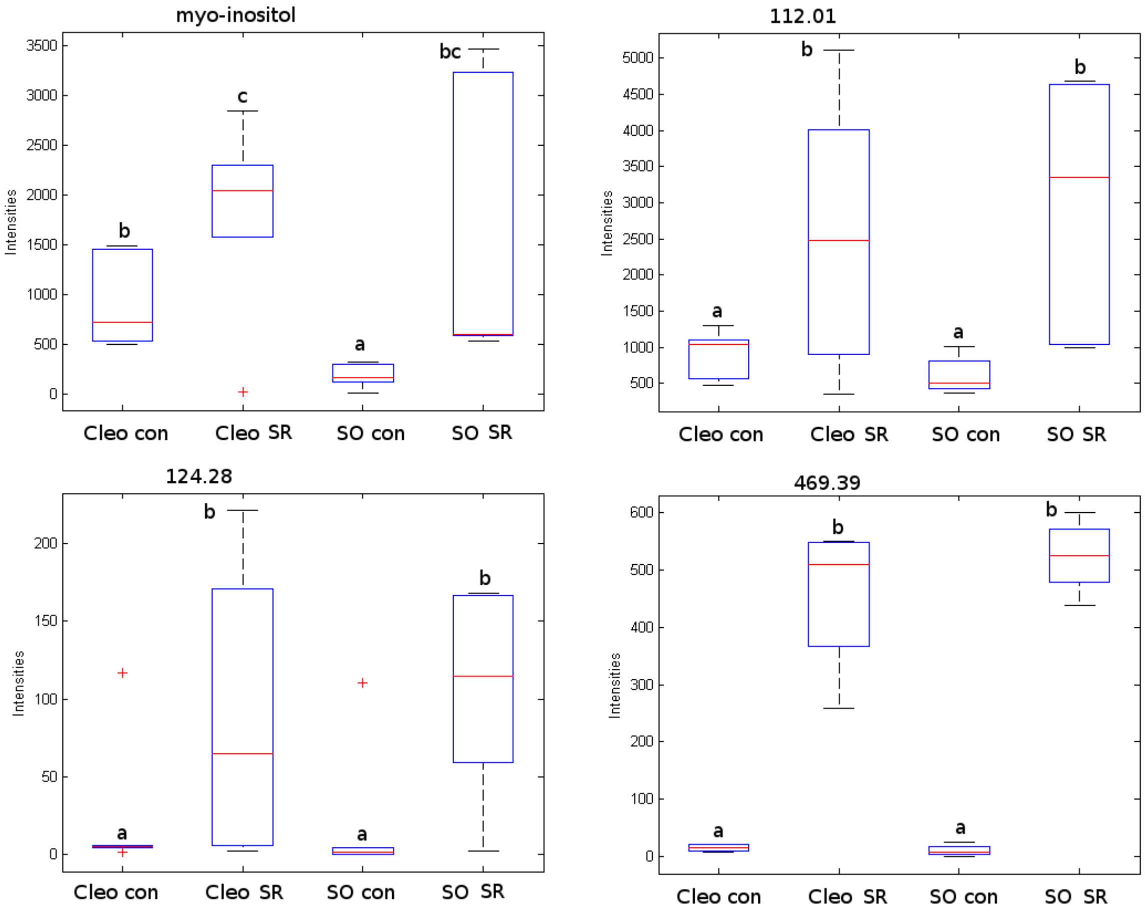

Supplementary Figure 1. Four signals accumulated in the leaf efflux of sour orange and Cleopatra mandarin SR plants. A signal was identified as myo-inositol and the others are unknown (112.01, 124.28 and 469.39 m/z). Boxplot analysis of the relative abundance of the four compounds in sour orange (SO) and Cleopatra mandarin (Cleo) either in the absence (con) or presence of infestation with spider mites (SR). Different letters indicate significant differences ($P < 0.05$; ANOVA) between treatments.

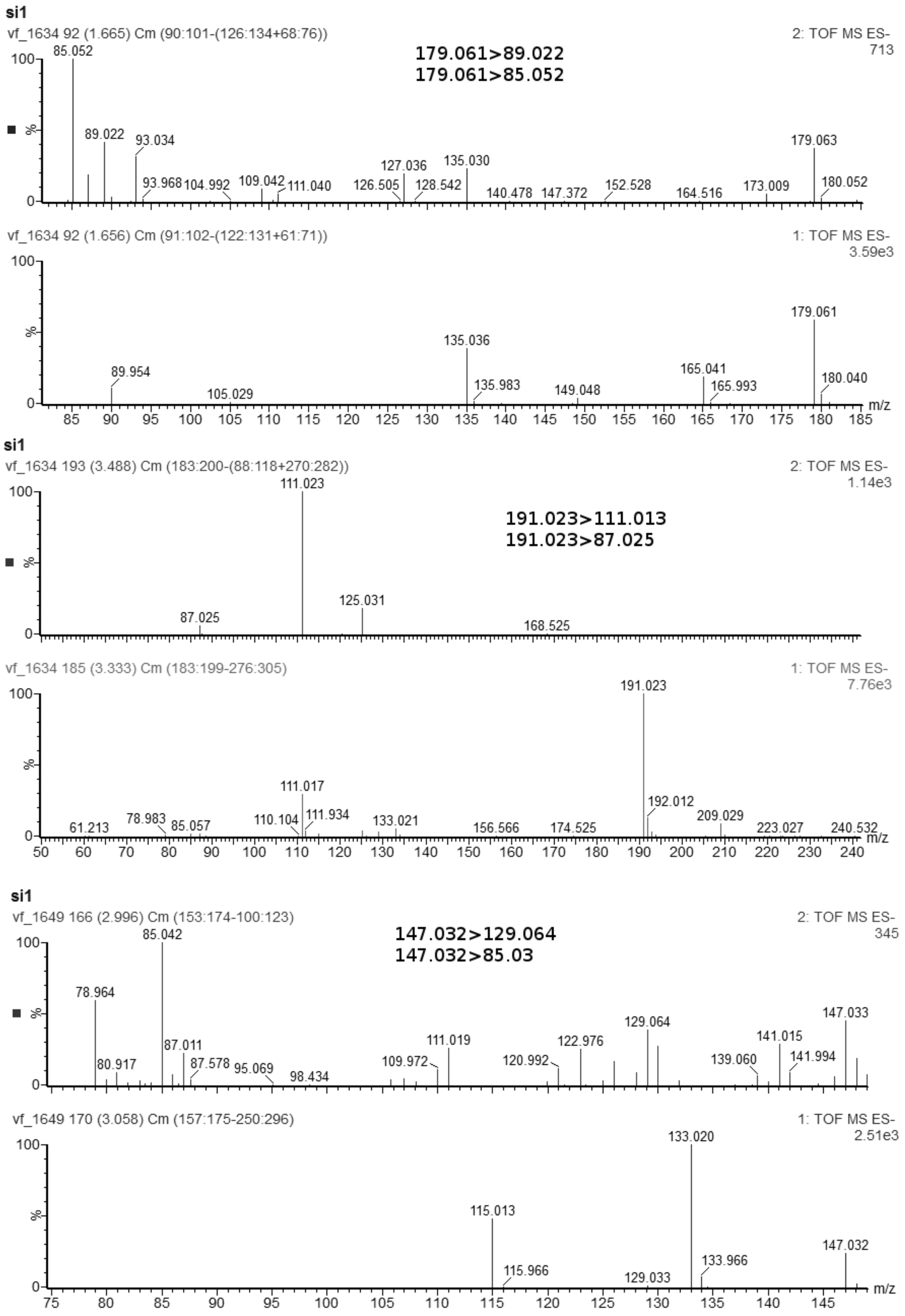

Supplementary figure 2. Identification of overaccumulated compounds. To determine the exact mass of the parental ion, no collision energy is applied (1 – TOF MS EI+/−). To obtain the fragmentation spectrum, a collision energy ramp from 5 to 45 eV is applied to the parental ion (2 – TOF MS EI +/−). Theoretical transitions were first checked in mass spectrum databases (Metlin).

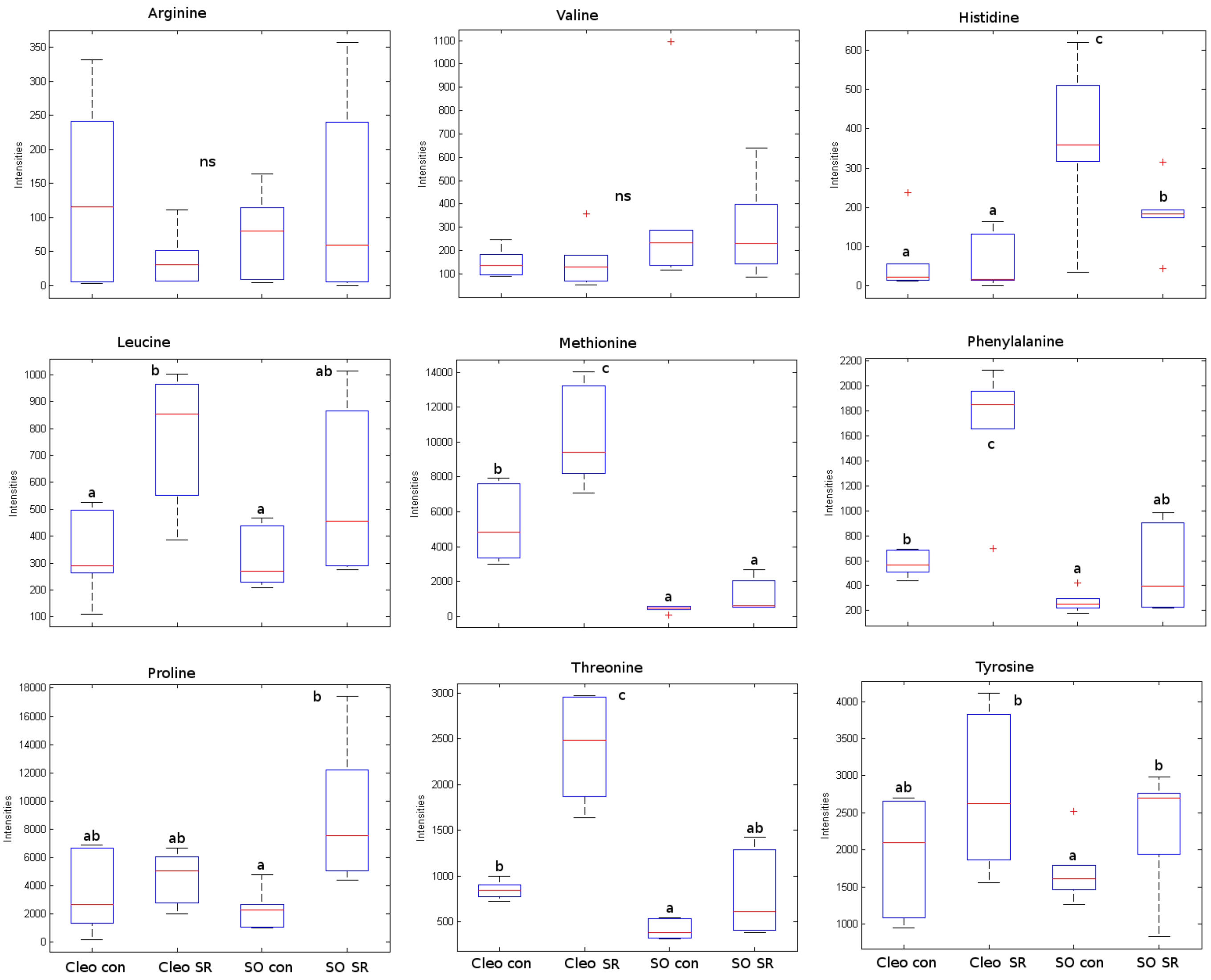

Supplementary figure 3. Amino acid profile in the leaf efflux in sour orange and Cleopatra mandarin following spider mite infestation. Uninfested sour orange (SO con), sour orange previously infested (SO SR), uninfested Cleopatra mandarin (Cleo con) and Cleopatra mandarin previously infested (Cleo SR). Twelve-week-old plants were infested with 10 mites per plant. Three days later, infested leaves were cut and we collected the leaf efflux in an EDTA solution over eight hours. The samples were quantified by HPLC-QTOFMS and were processed using an amino acid library. Boxplots represent the average of three independent experiments with two technical replicates (n=6). Different letters indicate significant differences (one-way ANOVA, $P < 0.05$; LSD) between treatments.

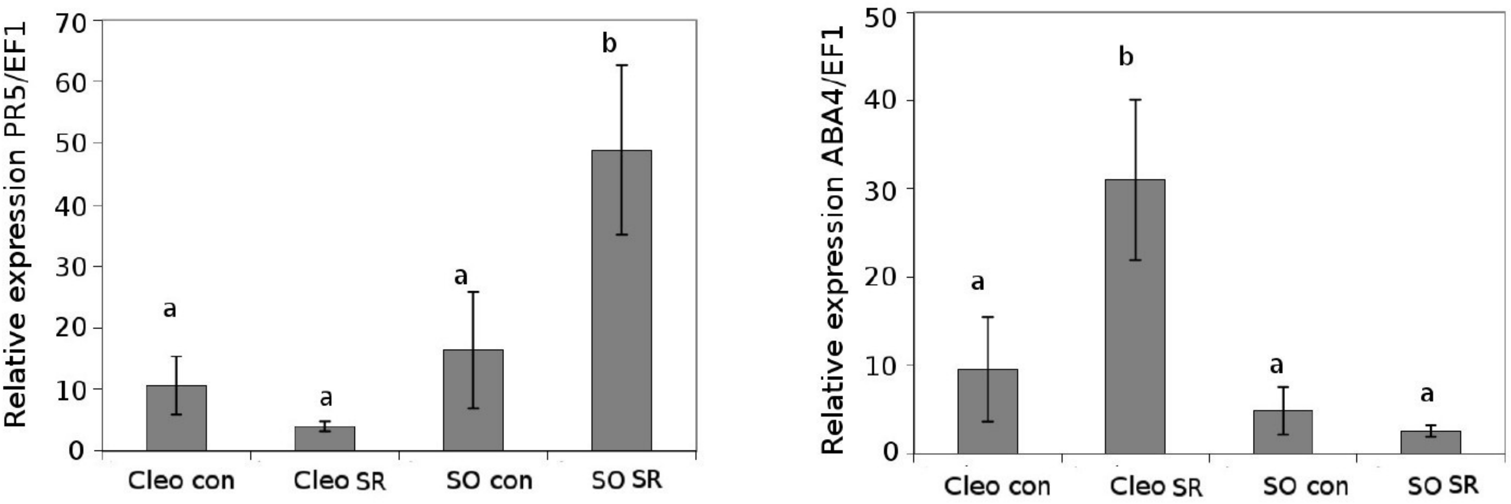

Supplementary figure 4. *PR5* andf *ABA4* expression in in sour orange (SO) and Cleopatra (Cleo) mandarin after SR treatment. Three days later, uninfested distal leaves of these plants were collected for mRNA analysis. Data are presented as a mean of three independent analyses of transcript expression relative to the housekeeping gene plants ± SD (n = 3). Different letters indicate significant differences (one-way ANOVA, $P < 0.05$; LSD) between treatments with Ct values as described by Yuan et al. (2006).

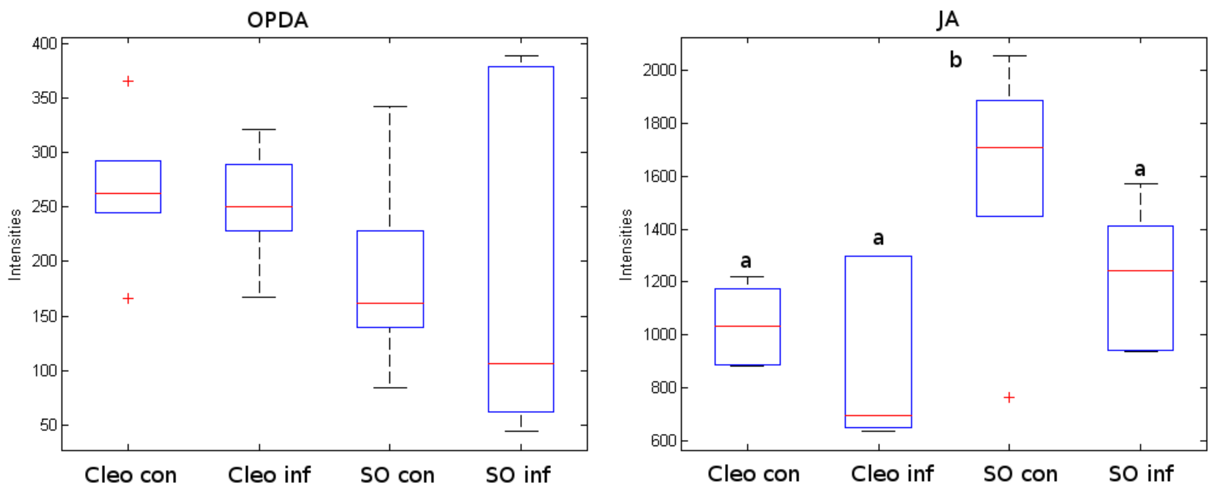

Supplementary figure 5. Hormonal profile in the root eflux in grafted plants following spider mite infestation. Clemenules variety grafted onto sour orange (SO) and Clemenules variety grafted onto Cleopatra mandarin (Cleo) plants uninfested (con) or infested (inf). Two year-old grafted plants were infested with 20 mites per plant. Three days later the stem was cut and the root efluxe was collected using a Scholander-type pressure chamber. The samples were quantified by HPLC-QTOFMS and were processed using a hormonal library. Boxplots represent the average of three independent experiments with two technical replicates (n=6). Different letters indicate significant differences (one-way ANOVA, P < 0.05; LSD) between treatments.

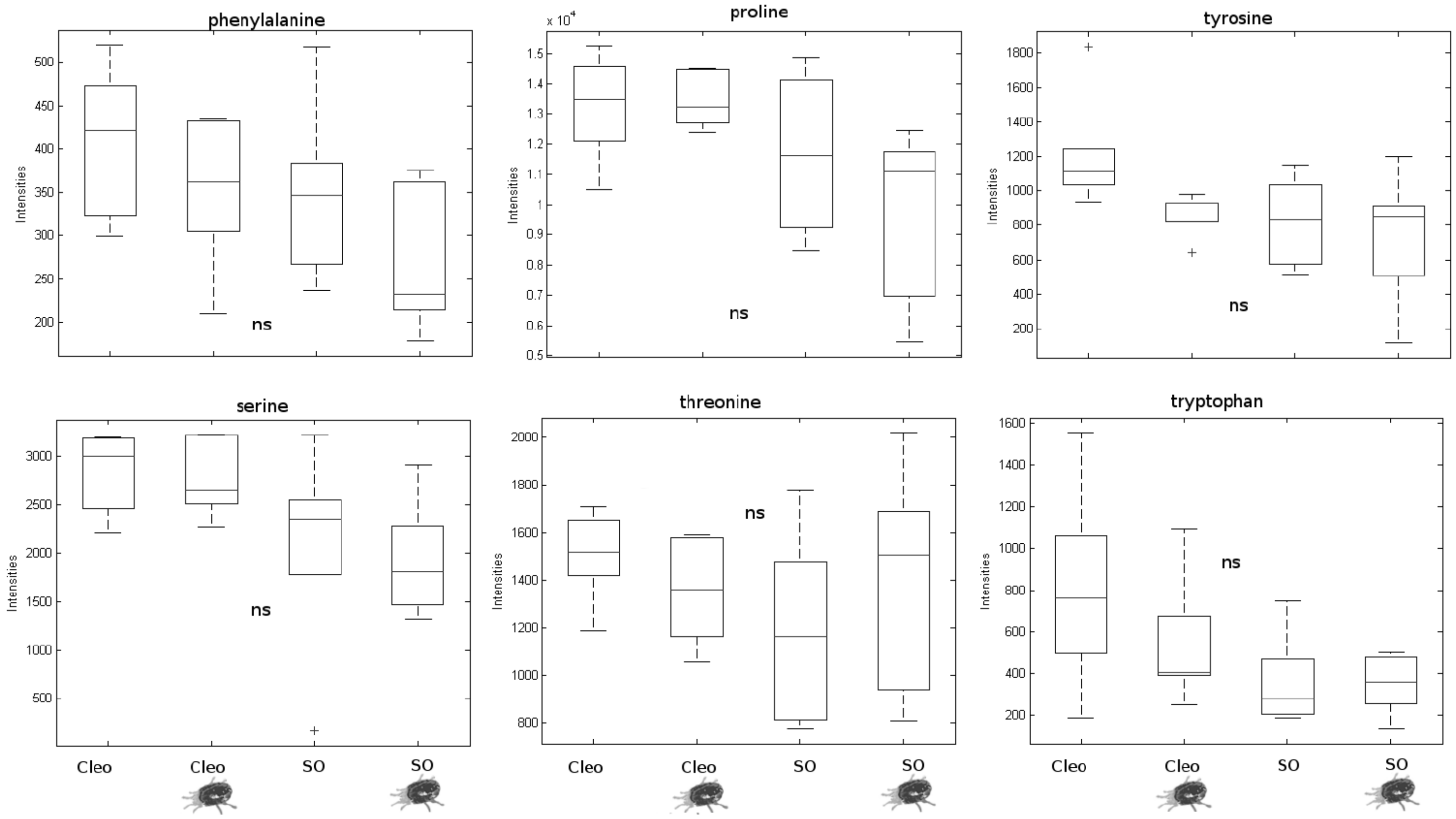

Supplementary figure 6. Amino acids showing non significant changes in the root efflux from rootstocks following spider mite infestation. Clemenules variety grafted onto sour orange (SO) and Clemenules variety grafted onto Cleopatra mandarin (Cleo) plants were either uninfested or infested (mite cartoon). Two year-old grafted plants were infested with 20 mites per plant. Three days later, the stem was cut and the root efflux was collected using a Scholander pressure chamber. The samples were quantified by HPLC-QTOFMS and were processed using an amino acid library. Boxplots represent the average of three independent experiments with two technical replicates (n=6). Different letters indicate significant differences (one-way ANOVA, $P < 0.05$; LSD) between treatments.
